# Plasma C-Reactive Protein and Clinical Outcomes after Acute Ischemic Stroke: A Prospective Observational Study

**DOI:** 10.1371/journal.pone.0156790

**Published:** 2016-06-03

**Authors:** Ryu Matsuo, Tetsuro Ago, Jun Hata, Yoshinobu Wakisaka, Junya Kuroda, Takahiro Kuwashiro, Takanari Kitazono, Masahiro Kamouchi, on behalf of the Fukuoka Stroke Registry Investigators

**Affiliations:** 1 Department of Medicine and Clinical Science, Graduate School of Medical Sciences, Kyushu University, Fukuoka, Japan; 2 Department of Health Care Administration and Management, Graduate School of Medical Sciences, Kyushu University, Fukuoka, Japan; 3 Center for Cohort Studies, Graduate School of Medical Sciences, Kyushu University, Fukuoka, Japan; 4 Department of Cerebrovascular Medicine and Neurology, Cerebrovascular Center and Clinical Research Institute, National Hospital Organization Kyushu Medical Center, Fukuoka, Japan; Federico II University of Naples, ITALY

## Abstract

**Background and Purpose:**

Although plasma C-reactive protein (CRP) is elevated in response to inflammation caused by brain infarction, the association of CRP with clinical outcomes after acute ischemic stroke remains uncertain. This study examined whether plasma high-sensitivity CRP (hsCRP) levels at onset were associated with clinical outcomes after acute ischemic stroke independent of conventional risk factors and acute infections after stroke.

**Methods:**

We prospectively included 3653 patients with first-ever ischemic stroke who had been functionally independent and were hospitalized within 24 h of onset. Plasma hsCRP levels were measured on admission and categorized into quartiles. The association between hsCRP levels and clinical outcomes, including neurological improvement, neurological deterioration, and poor functional outcome (modified Rankin scale ≥3 at 3 months), were investigated using a logistic regression analysis.

**Results:**

Higher hsCRP levels were significantly associated with unfavorable outcomes after adjusting for age, sex, baseline National Institutes of Health Stroke Scale score, stroke subtype, conventional risk factors, intravenous thrombolysis and endovascular therapy, and acute infections during hospitalization (multivariate-adjusted odds ratios [95% confidence interval] in the highest quartile versus the lowest quartile as a reference: 0.80 [0.65–0.97] for neurological improvement, 1.72 [1.26–2.34] for neurological deterioration, and 2.03 [1.55–2.67] for a poor functional outcome). These associations were unchanged after excluding patients with infectious diseases occurring during hospitalization, or those with stroke recurrence or death. These trends were similar irrespective of stroke subtypes or baseline stroke severity, but more marked in patients aged <70 years (P_heterogeneity_ = 0.001).

**Conclusions:**

High plasma hsCRP is independently associated with unfavorable clinical outcomes after acute ischemic stroke.

## Introduction

Inflammation plays a key role in the pathogenesis of cerebrovascular diseases via mechanisms including the development of atherosclerosis, plaque instability, and triggering of plaque rupture [1]. Among peripheral blood marker of inflammation, C-reactive protein (CRP), an acute-phase protein, is the most extensively used and established marker [2]. Previous studies suggested that elevation of serum or plasma high-sensitivity CRP (hsCRP) is a strong risk factor for future vascular events, including stroke, in the general population [3–9]. In stroke patients, higher CRP levels in the blood were also shown to be an independent risk factor of future vascular events [9,10–14] or mortality [10,11,13,15–17].

During the acute stage of ischemic stroke, CRP in the blood rises in response to inflammation caused by ischemic brain damage [1,10]. Therefore, plasma CRP levels are elevated owing to underlying vascular lesions as well as inflammation caused by brain infarction in patients with acute ischemic stroke. In a middle cerebral artery occlusion model of adult rats, administration of human CRP resulted in significantly larger cerebral infarcts than control subjects [18]. Further, administration of a specific small-molecule inhibitor of CRP to rats undergoing acute myocardial infarction abrogated the increase in infarct size and cardiac dysfunction produced by injection of human CRP [19]. These findings indicate that CRP itself may worsen ischemic damage. In patients with acute stroke, some studies showed that CRP was related to post-stroke functional outcome [20–22], whereas other groups reported that the association disappeared after adjusting for confounders [16,23,24]. Thus, the association between CRP in the blood and clinical outcomes remains inconclusive in patients with acute ischemic stroke.

To determine whether CRP in the blood is independently associated with short-term outcomes after stroke, we prospectively enrolled patients with first-ever acute ischemic stroke within 24 h of onset and measured plasma CRP on admission using a uniform method. A high-sensitivity test for CRP was used to evaluate accurate levels of CRP, even at low levels. The aim of this study was to elucidate whether plasma hsCRP levels within 24 h of onset were associated with neurological and functional outcomes after acute ischemic stroke independent of conventional risk factors and acute infections.

## Methods

### Study subjects

We conducted an hsCRP sub-study of the Fukuoka Stroke Registry (FSR). FSR is a multicenter hospital-based registry, in which acute stroke patients within 7 days of onset were enrolled (UMIN Clinical Trial Registry 000000800) [25,26]. Kyushu University Hospital and six stroke centers (National Hospital Organization Kyushu Medical Center, National Hospital Organization Fukuoka-Higashi Medical Center, Fukuoka Red Cross Hospital, St. Mary’s Hospital, Steel Memorial Yawata Hospital, and Japan Labor Health and Welfare Organization Kyushu Rosai Hospital) in Fukuoka, Japan, participate in this registry. The institutional review board of all hospitals approved the study protocols. Written informed consent was obtained from patients or their family members. Standardized instruments were used to collect demographic characteristics, co-morbidities, laboratory data, and medical histories of the patients.

Stroke was defined as a sudden onset of focal neurological deficits persisting for more than 24 h, and was classified into ischemic stroke, brain hemorrhage, subarachnoid hemorrhage, or other types of stroke using brain imaging data (computed tomography and/or magnetic resonance imaging). In this study, ischemic stroke was further classified into four subtypes according to TOAST criteria [27]: cardioembolic, large artery atherosclerosis, small vessel occlusion, and others.

During the study period from June 2007 to May 2014, 9199 Japanese patients were prospectively registered in the FSR database, of which 4867 patients were diagnosed as having ischemic stroke and hospitalized within 24 h of onset. Patients with impaired activities of daily living before stroke onset or no data on admission hsCRP values were further excluded, and 3653 patients were finally included in the analysis of neurological improvement or deterioration. For analysis of the functional outcome at 3 months after the stroke, 50 patients who could not be followed at 3 months after onset were excluded (S1 Fig).

### Clinical assessment

Hypertension was defined as systolic blood pressure ≥140 mm Hg or diastolic blood pressure ≥90 mm Hg in the chronic stage, or as a previous history of treatment with antihypertensive drugs. Dyslipidemia was defined as a low-density lipoprotein-cholesterol level ≥3.62 mmol/L, high-density lipoprotein-cholesterol level ≤1.03 mmol/L, triglyceride level ≥1.69 mmol/L, or a previous history of treatment with a lipid-lowering drug. Diabetes mellitus was determined by either the diagnostic criteria of the Japan Diabetes Society [28] in the chronic stage or based on a medical history of diabetes. Atrial fibrillation was diagnosed based on electrocardiographic findings on admission or during hospitalization, or a previous history of atrial fibrillation. Smoking was defined as current or former cigarette smoking, and alcohol intake was defined as habitual consumption of alcohol beverages before onset of stroke. Chronic kidney disease was defined as an estimated glomerular filtration rate (eGFR) <60 mL/min per 1.73 m^2^, in which eGFR was determined using the equation proposed by the Japanese Society of Nephrology as follows: eGFR (mL/min per 1.73 m^2^) = 194 × (serum creatinine [mg/dL])^1.094^ × age [year])^-0.287^ × 0.739 (if female) [29]. Body mass index was measured at admission.

Intravenous thrombolysis was performed using recombinant tissue plasminogen activator. Endovascular therapy included intra-arterial thrombolysis, endovascular thrombectomy, thromboaspiration, and angioplasty. Acute infection was defined as a complication of infectious disease including respiratory tract infection, urinary tract infection, sepsis, and other infections during hospitalization. Neurological severity was scaled by the National Institutes of Health Stroke Scale (NIHSS) score. NIHSS score was assessed on admission and at discharge. Functional outcome was assessed by the modified Rankin Scale (mRS). During hospitalization, the NIHSS and mRS were assessed by trained stroke neurologists. The mRS at the 3-month time point was evaluated by trained and certified research nurses through telephone assessment on the basis of a standardized structured questionnaire that was validated in a previous study to minimize the inter-rater variability [30].

### Measurement of plasma CRP

To measure plasma hsCRP levels, venous blood samples were collected from patients on the first admission day within 24 h after symptom onset, and were subsequently frozen and transferred to a single laboratory (SRL, Inc., Tokyo, Japan). Plasma hsCRP levels were measured with latex-enhanced nephelometry. Patients were classified into four groups according to the quartiles of values of hsCRP on admission.

### Study outcomes

Neurological improvement was defined as a ≥4-point decrease in the NIHSS score during hospitalization or 0 point at discharge [31]. Neurological deterioration was defined as a ≥2-point increase in the NIHSS score during hospitalization [32]. A poor functional outcome was defined as disability (mRS 3–5) or death (mRS 6). Mortality was defined as all causes of death.

### Statistical analysis

Baseline characteristics according to the plasma hsCRP quartiles were compared by analysis of variance, the Wilcoxon rank sum test, and logistic regression analysis as appropriate. Logistic regression analysis was also used to estimate multivariable-adjusted odds ratios and 95% confidence intervals for study outcomes. Multivariable model included age, sex, baseline NIHSS score as a continuous variable, stroke subtypes, hypertension, dyslipidemia, diabetes mellitus, atrial fibrillation, smoking, drinking, chronic kidney disease, body mass index, intravenous thrombolytic therapy and endovascular therapy, and acute infections [26]. P for heterogeneity was calculated by adding the interaction term of hsCRP quartile × subgroup variable in the model. Statistical analyses were performed using Stata 13 (StataCorp LP, College Station, TX, USA). Probability values of <0.05 were considered statistically significant.

## Results

### Plasma hsCRP levels on admission and background characteristics

The mean age of the study subjects was 70.8±12.2 years, and 1330 patients (36.4%) were women. The median of plasma hsCRP levels on admission was 1.23 mg/L (interquartile range, 0.50–4.69 mg/L).

Table 1 shows the clinical characteristics of patients according to the hsCRP quartiles. With the elevation of plasma hsCRP level, the age and the frequencies of hypertension, diabetes mellitus, atrial fibrillation, and chronic kidney disease increased, while prevalence of dyslipidemia and drinking decreased. The proportion of cardioembolism was higher as the plasma hsCRP level increased. The admission NIHSS score and the frequency of intravenous thrombolytic therapy and endovascular therapy were increased with an increase in plasma hsCRP values. During hospitalization, 500 patients developed infectious diseases (296 respiratory tract infections, 161 urinary tract infections, 28 sepsis, and 112 other infections). Acute infections were more prevalent in patients with higher levels of plasma hsCRP.

**Table 1:** Characteristics of patients.

	Q1, n = 910	Q2, n = 936	Q3, n = 898	Q4, n = 909	
	(hsCRP ≤0.50)	(0.50< hsCRP ≤1.25)	(1.25< hsCRP ≤4.70)	(hsCRP >4.70)	P for trend
**Age, y, mean±SD**	68.6±13.3	69.7±12.0	71.3±11.0	73.7±11.9	<0.001
**Female, n (%)**	347 (38.1)	331 (35.4)	321 (35.8)	331 (36.4)	0.51
**Risk factors, n (%)**					
**Hypertension**	651 (71.5)	735 (78.5)	736 (82.0)	754 (83.0)	<0.001
**Dyslipidemia**	440 (48.4)	504 (53.9)	480 (53.5)	392 (43.1)	0.03
**Diabetes mellitus**	247 (27.1)	265 (28.3)	294 (32.9)	274 (30.2)	0.04
**Atrial fibrillation**	179 (19.7)	224 (24.0)	261 (29.1)	352 (38.8)	<0.001
**Smoking**	482 (53.0)	528 (56.5)	509 (56.8)	487 (53.9)	0.67
**Drinking**	394 (43.3)	383 (41.0)	353 (39.3)	316 (34.9)	<0.001
**Chronic kidney diseases, n (%)**	236 (25.9)	282 (30.2)	303 (33.8)	385 (42.4)	<0.001
**Body mass index, kg/m**^**2**^, **mean±SD**	22.5±3.3	23.6±3.4	23.5±3.8	22.9±3.9	0.15
**Previous stroke, n (%)**	132 (14.5)	142 (15.2)	135 (15.0)	145 (16.0)	0.43
**Stroke subtype, n (%)**					
**Cardioembolism**	188 (20.7)	217 (23.2)	249 (27.7)	343 (37.7)	<0.001
**Large artery atherosclerosis**	109 (12.0)	149 (15.9)	172 (19.2)	160 (17.6)	
**Small vessel occlusion**	354 (38.9)	335 (35.8)	254 (28.3)	168 (18.5)	
**Others**	259 (28.5)	235 (25.1)	223 (24.8)	238 (26.2)	
**NIHSS score on admission, median (IQR)**	3 (1–5)	3 (2–5)	4 (2–8)	6 (3–13)	<0.001
**Thrombolytic and endovascular therapy, n (%)**					
**Intravenous thrombolysis**	110 (12.1)	116 (12.4)	114 (12.7)	146 (16.1)	0.02
**Endovascular therapy**	12 (1.3)	16 (1.7)	12 (1.3)	31 (3.4)	0.004
**Acute infections, n (%)**	63 (6.9)	89 (9.5)	112 (12.5)	236 (26.0)	<0.001

NIHSS: National Institutes of Health Stroke Scale, IQR: interquartile range. Q1–Q4 indicate four groups according to the quartile of hsCRP values (mg/L).

### Association between plasma hsCRP levels on admission and clinical outcomes

The odds ratio for neurological improvement decreased as the level of plasma hsCRP increased after adjustment for age, sex, baseline neurological severity, and stroke subtypes (Table 2). Further adjustment for conventional risk factors and acute infections did not alter the results.

**Table 2:** Plasma hsCRP levels and neurological improvement during hospitalization.

		Age- and sex-adjusted		Multivariable-adjusted (model 1)		Multivariable-adjusted (model 2)	
	Events(%)	OR (95% CI)	P	OR (95% CI)	P	OR (95% CI)	P
**Q1, n = 910 (hsCRP ≤0.50)**	498 (54.7)	1.00 (reference)		1.00 (reference)		1.00 (reference)	
**Q2, n = 936 (0.50< hsCRP ≤1.25)**	467 (49.9)	0.83 (0.69–1.00)	0.047	0.83 (0.69–1.00)	0.06	0.83 (0.67–1.01)	0.05
**Q3, n = 898 (1.25< hsCRP ≤4.70)**	445 (49.6)	0.83 (0.69–1.00)	0.047	0.78 (0.65–0.95)	0.01	0.82 (0.68–0.99)	0.048
**Q4, n = 909 (hsCRP >4.70)**	444 (48.8)	0.82 (0.68–0.99)	0.04	0.69 (0.57–0.84)	<0.001	0.80 (0.65–0.97)	0.03
**P for trend**			0.046		<0.001		0.03

OR: odds ratio, CI: confidence interval. Q1–Q4 indicate four groups according to the quartile of hsCRP values (mg/L). Model 1 included age, sex, baseline National Institutes of Health Stroke Scale score, and stroke subtypes. Model 2 included the same variables as those in model 1, and hypertension, dyslipidemia, diabetes mellitus, atrial fibrillation, smoking, drinking, chronic kidney disease, body mass index, intravenous thrombolytic therapy and endovascular therapy, and acute infections.

Similarly, higher levels of plasma hsCRP were significantly associated with an increased risk of neurological deterioration even after adjusting for multiple confounders (Table 3).

**Table 3:** Plasma hsCRP levels and neurological deterioration during hospitalization.

		Age- and sex-adjusted		Multivariable-adjusted (model 1)		Multivariable-adjusted (model 2)	
	Events (%)	OR (95% CI)	P	OR (95% CI)	P	OR (95% CI)	P
**Q1, n = 910 (hsCRP ≤0.50)**	82 (9.0)	1.00 (reference)		1.00 (reference)		1.00 (reference)	
**Q2, n = 936 (0.50< hsCRP ≤1.25)**	101 (10.8)	1.21 (0.89–1.64)	0.23	1.08 (0.79–1.49)	0.62	1.20 (0.88–1.66)	0.25
**Q3, n = 898 (1.25< hsCRP ≤4.70)**	130 (14.5)	1.64 (1.22–2.21)	0.001	1.47 (1.08–2.00)	0.01	1.57 (1.15–2.14)	0.004
**Q4, n = 909 (hsCRP >4.70)**	171 (18.8)	2.14 (1.61–2.85)	<0.001	1.94 (1.43–2.62)	<0.001	1.72 (1.26–2.34)	0.001
**P for trend**			<0.001		<0.001		<0.001

OR: odds ratio, CI: confidence interval. Q1–Q4 indicate four groups according to the quartile of hsCRP values (mg/L). Model 1 included age, sex, baseline National Institutes of Health Stroke Scale score, and stroke subtypes. Model 2 included the same variables as those in model 1, and hypertension, dyslipidemia, diabetes mellitus, atrial fibrillation, smoking, drinking, chronic kidney disease, body mass index, intravenous thrombolytic therapy and endovascular therapy, and acute infections.

The plasma hsCRP levels showed a significant association with poor functional outcome at discharge and at 3 months (Table 4).

**Table 4:** Plasma hsCRP levels and poor functional outcome at discharge and at 3 months.

		Age- and sex-adjusted		Multivariable-adjusted (model 1)		Multivariable-adjusted (model 2)	
	Events (%)	OR (95% CI)	P	OR (95% CI)	P	OR (95% CI)	P
**At discharge**							
**Q1, n = 910 (hsCRP ≤0.50)**	191 (21.0)	1.00 (reference)		1.00 (reference)		1.00 (reference)	
**Q2, n = 936 (0.50< hsCRP ≤1.25)**	245 (26.2)	1.33 (1.06–1.66)	0.01	1.28 (0.99–1.65)	0.06	1.33 (1.02–1.73)	0.04
**Q3, n = 898 (1.25< hsCRP ≤4.70)**	331 (36.9)	2.12 (1.71–2.63)	<0.001	1.74 (1.35–2.25)	<0.001	1.72 (1.33–2.24)	<0.001
**Q4, n = 909 (hsCRP >4.70)**	493 (54.2)	4.05 (3.27–5.01)	<0.001	2.85 (2.21–3.67)	<0.001	2.35 (1.81–3.06)	<0.001
**P for trend**			<0.001		<0.001		<0.001
**At 3 months**							
**Q1, n = 896 (hsCRP ≤0.50)**	166 (18.5)	1.00 (reference)		1.00 (reference)		1.00 (reference)	
**Q2, n = 928 (0.50< hsCRP ≤1.25)**	198 (21.3)	1.18 (0.93–1.50)	0.17	1.08 (0.82–1.41)	0.60	1.09 (0.83–1.45)	0.53
**Q3, n = 885 (1.25< hsCRP ≤4.70)**	282 (31.9)	1.98 (1.58–2.49)	<0.001	1.52 (1.17–1.99)	0.002	1.56 (1.19–2.06)	0.001
**Q4, n = 894 (hsCRP >4.70)**	439 (49.1)	3.79 (3.03–4.74)	<0.001	2.47 (1.90–3.21)	<0.001	2.03 (1.55–2.67)	<0.001
**P for trend**			<0.001		<0.001		<0.001

OR: odds ratio, CI: confidence interval. Q1–Q4 indicate four groups according to the quartile of hsCRP values (mg/L). Model 1 included age, sex, baseline National Institutes of Health Stroke Scale score, and stroke subtypes. Model 2 included the same variables as those in model 1, and hypertension, dyslipidemia, diabetes mellitus, atrial fibrillation, smoking, drinking, chronic kidney disease, body mass index, intravenous thrombolytic therapy and endovascular therapy, and acute infections.

For sensitivity analyses, we analyzed the associations in patients who did not experience acute infections after stroke onset (Table 5). However, the associations between plasma hsCRP levels and clinical outcomes were still observed.

**Table 5:** Plasma hsCRP levels and clinical outcomes in patients without post-stroke acute infections.

	Neurological improvement			Neurological deterioration			Poor functional outcome		
	Events/number (%)	Multivariable-adjusted OR (95% CI)	P	Events/number (%)	Multivariable-adjusted OR (95% CI)	P	Events/number (%)	Multivariable-adjusted OR (95% CI)	P
**Q1 (hsCRP ≤0.46)**	426/780 (55.3)	1.00 (reference)		52/780 (6.7)	1.00 (reference)		107/771 (13.9)	1.00 (reference)	
**Q2 (0.46< hsCRP ≤1.10)**	396/789 (50.8)	0.85 (0.69–1.05)	0.12	65/789 (8.3)	1.05 (0.71–1.57)	0.81	131/780 (16.8)	1.04 (0.75–1.44)	0.82
**Q3 (1.10< hsCRP ≤3.68)**	403/794 (51.4)	0.88 (0.71–1.08)	0.23	83/794 (10.6)	1.44 (0.98–2.12)	0.06	180/784 (23.0)	1.47 (1.08–2.00)	0.02
**Q4 (hsCRP >3.68)**	402/790 (51.9)	0.79 (0.63–0.98)	0.03	110/790 (14.2)	2.04 (1.40–2.97)	<0.001	296/774 (38.2)	2.28 (1.69–3.09)	<0.001
**P for trend**			0.06			<0.001			<0.001

OR: odds ratio, CI: confidence interval. Q1–Q4 indicate four groups according to the quartile of hsCRP values (mg/L). Functional outcome was assessed at 3 months after stroke onset. Multivariable model included age, sex, baseline National Institutes of Health Stroke Scale score, stroke subtypes, hypertension, dyslipidemia, diabetes mellitus, atrial fibrillation, smoking, drinking, chronic kidney disease, body mass index, intravenous thrombolytic therapy and endovascular therapy, and acute infections.

Plasma hsCRP levels were not associated with early recurrence in this study (S1 Table). However, to exclude the possibility that the association was affected by early stroke recurrence or mortality, we performed additional analysis after excluding 220 patients who experienced recurrent stroke and 129 patients who died at 3 months after the onset. However, the trend was essentially unchanged (S2 Table).

### Subgroup analysis

**Fig 1:**
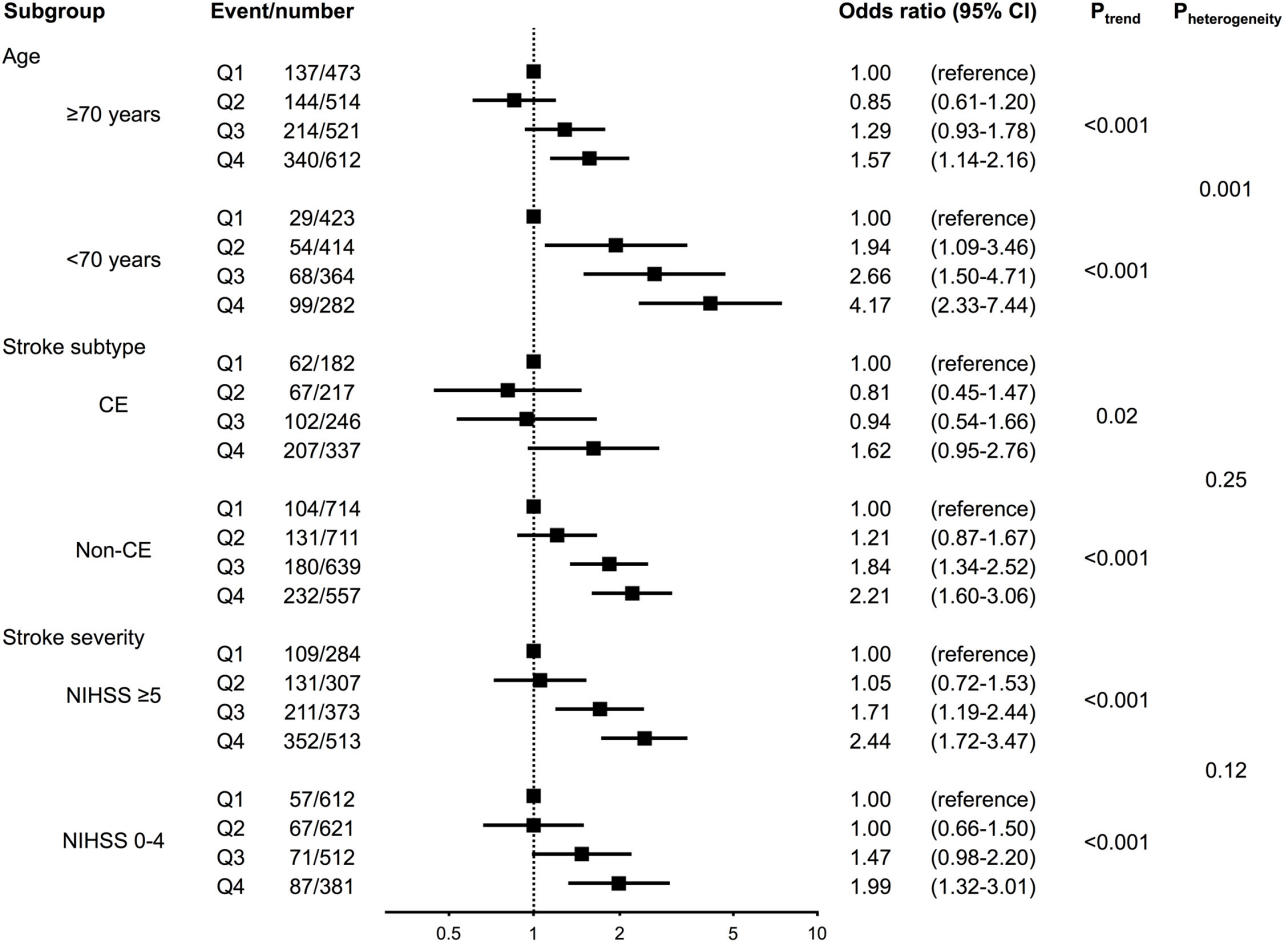
Subgroup analysis. Multivariable-adjusted odds ratio and 95% confidence interval of each hsCRP quartile for poor functional outcome at 3 months are shown according to subgroups. Q1–Q4 indicate the four groups according to the quartile of hsCRP (mg/L). The multivariable model included age, sex, baseline NIHSS score, stroke subtypes, hypertension, dyslipidemia, diabetes mellitus, atrial fibrillation, smoking, drinking, chronic kidney disease, body mass index, intravenous thrombolytic therapy and endovascular therapy, and acute infections. Subgroups included age (≥70 years or <70 years), stroke subtypes (cardioembolic or others), and stroke severity (baseline NIHSS score 0–4 or NIHSS score ≥5). P for heterogeneity (P_heterogeneity_) was calculated by means of the interaction term.

The association between plasma hsCRP levels and poor functional outcome at 3 months was then investigated according to baseline characteristics (Fig 1). The trends were maintained irrespective of stroke subtype and initial neurological severity; however, odds ratios for poor functional outcome increased more markedly as an elevation of plasma hsCRP levels in patients aged <70 years than in those aged ≥70 years (P for heterogeneity = 0.001). The heterogeneity between the two age groups was still found even after excluding patients with acute infections during hospitalization (P for heterogeneity = 0.003, S2 Fig). There was no heterogeneity between the two groups for stroke subtype and stroke severity.

## Discussion

The present study showed that elevated plasma hsCRP levels within 24 h of onset were associated with poor short-term clinical outcomes, including neurological deterioration and poor functional outcome, in patients with acute ischemic stroke. Our findings indicate that plasma CRP was independently associated with unfavorable clinical outcomes after acute ischemic stroke.

The association between CRP in the blood and post-stroke clinical outcomes is still unclear. Although some groups showed a positive association [20–22], the number of the patients was limited, and the influence of confounding factors was not controlled. In fact, other studies reported that the association of CRP with clinical outcomes (3-month mortality [23], new vascular events [16], or stroke recurrence or death within 90 days [24]) disappeared after adjusting for confounders. In our study, however, the association was unaffected by further adjustment for potentially confounding factors, including age, vascular risk factors, stroke subtype, baseline severity, and acute infections during hospitalization. Additionally, the trends were unchanged even after excluding patients with infectious diseases occurring during hospitalization or those who experienced recurrent stroke or died within 3 months after stroke onset. Further, plasma hsCRP had a more significant impact on clinical outcomes in younger than older patients. Plasma hsCRP levels increase with age, and post-stroke functional outcome is generally poor in aged patients. Therefore, the association may be weakened by these factors in older patients. These findings are consistent with the concept that plasma hsCRP is a factor related to unfavorable outcomes independent of conventional risk factors and acute infections in patients with acute ischemic stroke.

CRP belongs to the pentraxin family of calcium-dependent ligand-binding plasma proteins. Human CRP is predominantly produced in hepatocytes in response to proinflammatory cytokines such as interleukin-6, and binds phosphocholine residues in a variety of autologous ligands, which is considered to be related to innate immunity in humans [1,33–35]. Although the precise mechanisms underlying the association between plasma hsCRP and clinical outcomes after ischemic stroke are unclear, there are two possible explanations. The first involves exacerbation of brain infarction, as CRP itself may worsen clinical outcomes after ischemic stroke. However, it remains unclear whether inhibition of the protein can ameliorate clinical outcomes in acute ischemic stroke patients. Alternatively, assuming that CRP plays a beneficial rather than detrimental role via clearance of apoptotic and necrotic cells [1,35], plasma CRP may be elevated to counteract an exacerbating factor. Indeed, a recent study in which pure natural human CRP was administered to healthy adult human volunteers reported no proinflammatory effects [36]. Moreover, atherosclerosis was not reduced in CRP-deficient mice, providing evidence against a proatherogenic role of CRP [37]. Further studies are required to elucidate the pathophysiological role of plasma CRP in acute ischemic stroke.

The number of patients in our study was very large, enabling stratified analysis and adjustment for potential confounders. Further, even low levels of plasma CRP could be measured by high-sensitivity assays using a standardized method. However, a limitation of our study was that the exact timing of collecting the blood samples was unknown, even if it were collected within 24 h. Thus, the time elapsed from stroke onset could not be adjusted for. Nevertheless, patients with severe stroke are likely to be hospitalized early, when CRP levels are still on the rise. Therefore, this may weaken the association. Our results may also be biased by unidentified confounders that were not adjusted for. Further, although we found no sex difference in clinical outcomes (P for heterogeneity >0.07) in this study, the proportion of female individuals was small. Our findings were only in patients included in the restricted region, and cannot be generalized to the larger population. Thus, associations between plasma CRP and short-term clinical outcomes after ischemic stroke should be further validated in other cohorts.

## Supporting Information

S1 Fig Flow chart of patient selection. Selection of patients for each analysis is shown. FSR: Fukuoka Stroke Registry, ADL: activities of daily living.
(DOCX)

S2 Fig Subgroup analysis in patients without post-stroke acute infections. Multivariable-adjusted odds ratio and 95% confidence interval of each hsCRP quartile for poor functional outcome at 3 months in patients without acute infections during hospitalization are shown according to subgroups. Q1–Q4 indicate the four groups according to the quartile of hsCRP values (mg/L). Subgroups include age (≥70 years or <70 years), stroke subtypes (cardioembolic [CE] or others), and stroke severity (baseline NIHSS score 0–4 or NIHSS score ≥5). Multivariable model included age, sex, baseline National Institutes of Health Stroke Scale (NIHSS) score, stroke subtypes, hypertension, dyslipidemia, diabetes mellitus, atrial fibrillation, smoking, drinking, chronic kidney disease, body mass index, and intravenous thrombolytic therapy and endovascular therapy. In subgroup analysis for age and stroke severity, these variables were included in the model as dichotomized values. P for heterogeneity (P_heterogeneity_) was calculated by means of the interaction term.
(DOCX)

S1 Table Plasma hsCRP levels and stroke recurrence during hospitalization. OR: odds ratio, CI: confidence interval. Q1–Q4 indicate the four groups according to the quartile of hsCRP values (mg/L). Model 1 included age, sex, baseline National Institutes of Health Stroke Scale score, and stroke subtypes.
(DOCX)

S2 Table Plasma hsCRP levels and clinical outcomes in patients without stroke recurrence or death within 3 months. OR: odds ratio, CI: confidence interval. Q1–Q4 indicate the four groups according to the quartile of hsCRP values (mg/L). Functional outcome was assessed at 3 months after stroke onset. Multivariable model included age, sex, baseline National Institutes of Health Stroke Scale score, stroke subtypes, hypertension, dyslipidemia, diabetes mellitus, atrial fibrillation, smoking, drinking, chronic kidney disease, body mass index, intravenous thrombolytic therapy and endovascular therapy, and acute infections.
(DOCX)
