# Supplementary material for: Plasma C-Reactive Protein and Clinical Outcomes after Acute Ischemic Stroke: A Prospective Observational Study
Source: PLoS One. 2016 Jun 3;11(6):e0156790. doi: 10.1371/journal.pone.0156790 (PMC4892536; doi:10.1371/journal.pone.0156790)
Supplement: S1 Fig — Selection of patients for each analysis is shown. FSR: Fukuoka Stroke Registry, ADL: activities of daily living. (DOCX) [file pone.0156790.s001.docx]

**S1 Fig. Flow chart of patient selection.**

Selection of patients for each analysis is shown. FSR: Fukuoka Stroke Registry, ADL: activities of daily living, CRP: C-reactive protein.
