# Supplementary material for: Plasma C-Reactive Protein and Clinical Outcomes after Acute Ischemic Stroke: A Prospective Observational Study
Source: PLoS One. 2016 Jun 3;11(6):e0156790. doi: 10.1371/journal.pone.0156790 (PMC4892536; doi:10.1371/journal.pone.0156790)
Supplement: S2 Fig — Multivariable-adjusted odds ratio and 95% confidence interval of each hsCRP quartile for poor functional outcome at 3 months in patients without acute infections during hospitalization are shown according to subgroups. Q1–Q4 indicate the four groups according to the quartile of hsCRP values (mg/L). Subgroups include age (≥70 years or <70 years), stroke subtypes (cardioembolic [CE] or others), and stroke severity (baseline NIHSS score 0–4 or NIHSS score ≥5). Multivariable model included age, sex, baseline National Institutes of Health Stroke Scale (NIHSS) score, stroke subtypes, hypertension, dyslipidemia, diabetes mellitus, atrial fibrillation, smoking, drinking, chronic kidney disease, body mass index, and intravenous thrombolytic therapy and endovascular therapy. In subgroup analysis for age and stroke severity, these variables were included in the model as dichotomized values. P for heterogeneity (Pheterogeneity) was calculated by means of the interaction term. (DOCX) [file pone.0156790.s002.docx]

**S2 Fig. Subgroup analysis in patients without post-stroke acute infections.**

Multivariable-adjusted odds ratio and 95% confidence interval of each hsCRP quartile for poor functional outcome at 3 months in patients without acute infections during hospitalization are shown according to subgroups. Q1–Q4 indicate four groups according to the quartile of hsCRP values (mg/L). Multivariable model included age, sex, baseline National Institutes of Health Stroke Scale (NIHSS) score, stroke subtypes, hypertension, dyslipidemia, diabetes mellitus, atrial fibrillation, smoking, drinking, chronic kidney disease, body mass index, and intravenous thrombolytic therapy and endovascular therapy. Subgroups include age (≥70 years or <70 years), stroke subtypes (cardioembolic [CE] or others), and stroke severity (baseline NIHSS score 0–4 or NIHSS score ≥5). P for heterogeneity (P_heterogeneity_) was calculated by means of the interaction term.
