# Supplementary material for: Plasma C-Reactive Protein and Clinical Outcomes after Acute Ischemic Stroke: A Prospective Observational Study
Source: PLoS One. 2016 Jun 3;11(6):e0156790. doi: 10.1371/journal.pone.0156790 (PMC4892536; doi:10.1371/journal.pone.0156790)
Supplement: S1 Table — OR: odds ratio, CI: confidence interval. Q1–Q4 indicate the four groups according to the quartile of hsCRP values (mg/L). Model 1 included age, sex, baseline National Institutes of Health Stroke Scale score, and stroke subtypes. (DOCX) [file pone.0156790.s003.docx]

**S1 Table. Plasma hsCRP levels and stroke recurrence during hospitalization.**

|  |  |  | Age- and sex-adjusted | |  | Multivariable-adjusted  (model 1) | |
| --- | --- | --- | --- | --- | --- | --- | --- |
|  | Events (%) |  | OR (95% CI) | P |  | OR (95% CI) | P |
| Q1, n=910  (hsCRP ≤0.50) | 29 (3.2) |  | 1.00 (reference) |  |  | 1.00 (reference) |  |
| Q2, n=936  (0.50< hsCRP ≤1.25) | 36 (3.9) |  | 1.19 (0.72–1.96) | 0.49 |  | 1.15(0.69–1.90) | 0.59 |
| Q3, n=898  (1.25< hsCRP ≤4.70) | 46 (5.1) |  | 1.56 (0.97–2.51) | 0.07 |  | 1.36 (0.84–2.21) | 0.21 |
| Q4, n=909  (hsCRP >4.70) | 43 (4.7) |  | 1.35 (0.83–2.20) | 0.22 |  | 1.08 (0.66–1.78) | 0.76 |
| P for trend |  |  |  | 0.14 |  |  | 0.66 |

OR: odds ratio, CI: confidence interval. Q1–Q4 indicate four groups according to the quartile of hsCRP values (mg/L). Model 1 included age, sex, baseline National Institutes of Health Stroke Scale score, and stroke subtypes.
