# Supplementary material for: Plasma C-Reactive Protein and Clinical Outcomes after Acute Ischemic Stroke: A Prospective Observational Study
Source: PLoS One. 2016 Jun 3;11(6):e0156790. doi: 10.1371/journal.pone.0156790 (PMC4892536; doi:10.1371/journal.pone.0156790)
Supplement: S2 Table — OR: odds ratio, CI: confidence interval. Q1–Q4 indicate the four groups according to the quartile of hsCRP values (mg/L). Functional outcome was assessed at 3 months after stroke onset. Multivariable model included age, sex, baseline National Institutes of Health Stroke Scale score, stroke subtypes, hypertension, dyslipidemia, diabetes mellitus, atrial fibrillation, smoking, drinking, chronic kidney disease, body mass index, intravenous thrombolytic therapy and endovascular therapy, and acute infections. (DOCX) [file pone.0156790.s004.docx]

**S2 Table. Plasma hsCRP levels and clinical outcomes in patients without stroke recurrence or death within 3 months.**

|  | Neurological improvement | | |  | Neurological deterioration | | |  | Poor functional outcome | | |
| --- | --- | --- | --- | --- | --- | --- | --- | --- | --- | --- | --- |
|  | Events/  number  (%) | Multivariable  -adjusted OR  (95% CI) | P |  | Events/  number  (%) | Multivariable  -adjusted OR  (95% CI) | P |  | Events/  number  (%) | Multivariable  -adjusted OR  (95% CI) | P |
| Q1  (hsCRP ≤0.49) | 453/824  (55.0) | 1.00  (reference) |  |  | 61/824  (7.4) | 1.00  (reference) |  |  | 131/824  (15.9) | 1.00  (reference) |  |
| Q2  (0.49< hsCRP ≤1.19) | 422/823  (51.3) | 0.88  (0.72–1.08) | 0.23 |  | 73/823  (8.9) | 0.99  (0.67–1.46) | 0.97 |  | 155/823  (18.8) | 0.98  (0.72–1.34) | 0.92 |
| Q3  (1.19< hsCRP ≤4.31) | 419/817  (51.3) | 0.86  (0.70–1.05) | 0.14 |  | 94/817  (11.5) | 1.41  (0.97–2.04) | 0.07 |  | 227/817  (27.8) | 1.45  (1.08–1.97) | 0.02 |
| Q4  (hsCRP >4.31) | 435/820  (53.0) | 0.83  (0.67–1.03) | 0.10 |  | 112/820  (13.7) | 1.68  (1.16–2.44) | 0.006 |  | 356/820  (43.4) | 1.84  (1.37–2.47) | <0.001 |
| P for trend |  |  | 0.09 |  |  |  | 0.001 |  |  |  | <0.001 |

OR: odds ratio, CI: confidence interval. Q1–Q4 indicate four groups according to the quartile of hsCRP values (mg/L). Functional outcome was assessed at 3 months after stroke onset. Multivariable model included age, sex, baseline National Institutes of Health Stroke Scale score, stroke subtypes, hypertension, dyslipidemia, diabetes mellitus, atrial fibrillation, smoking, drinking, chronic kidney disease, body mass index, intravenous thrombolytic therapy and endovascular therapy, and acute infections.
